# A new predictor of bleeding based on ultrasonographic features in percutaneous liver mass biopsy

**DOI:** 10.3906/sag-2005-338

**Published:** 2020-12-17

**Authors:** Ömer Faruk ATEŞ, Onur TAYDAŞ, Ahmet Burak KARA, Mehmet GÖKTEPELİ, Mustafa ÖZDEMİR

**Affiliations:** 1 Department of Radiology, Faculty of Medicine, Sakarya University, Sakarya Turkey; 2 Department of Radiology, Faculty of Medicine, Erzincan Binali Yıldırım University, Erzincan Turkey; 3 Department of Radiology, Kayseri City Hospital, Kayseri Turkey

**Keywords:** Biopsy, bleeding, liver, ultrasound

## Abstract

**Background/aim:**

An ultrasound-guided liver mass biopsy is a method frequently used in determining the diagnosis and treatment plan. The aim of this study was to evaluate the potential new predictors of bleeding based on ultrasonographic features in liver mass biopsies, which are frequently applied in routine clinical practice.

**Materials and methods:**

The images and data of patients aged over 18 years, who underwent an imaging-guided percutaneous liver mass biopsy between January 2018 and December 2019 with various indications, were retrospectively reviewed. Liver size, liver steatosis status, parenchyma appearance, and mass vascularity on Doppler ultrasonography before the procedure, and hemoglobin (Hb) values before and after the procedure were recorded.

**Results:**

A total of 176 patients were included in the study. Ninety-six patients were male (54.5%) and 80 were female (45.5%). The mean age of the patients was 64 ± 12.3 years. The mean hemoglobin values of the patients were 11.5 ± 1.9 gr/dL before the procedure and 11.4 ± 1.5 gr/dL after the procedure. While 144 of the patients had less than 10% hemoglobin decrease (81.8%), 32 had more than 10% decrease (8.2%). In 56 patients, a heterogeneous and coarse granular pattern was observed in the liver parenchyma (31.8%). The decrease in the Hb rate was significantly higher in patients with heterogeneous and coarse granular liver parenchyma (8.7%) than in patients with normal parenchyma (6.6%) (P = 0.036).

**Conclusion:**

In our study, it was shown for the first time in the literature that the ultrasonographic appearance of the liver (heterogeneous and coarse granular parenchyma) may also be one of the parameters that can help to predict the risk of bleeding.

## 1. Introduction

An ultrasound-guided liver mass biopsy is a method frequently used in determining the diagnosis and treatment plan, as liver primary neoplasms are common and the liver is one of the most frequently metastasized organs. Although it is a very reliable and effective method, there are also a number of complications, most important example of which is albeit rarely mortal complication causing hemorrhage. Although major bleeding complication has been reported rarely in ultrasound-guided liver biopsies [1–3], it can be particularly important in patients with comorbid diseases or underlying anemia. For this reason, it is important to determine the potential factors that increase or decrease the bleeding complication and assess the risk before the procedure. Some of the risk factors associated with bleeding risk have been reported in previous studies as subcapsular lesions [4], biopsy in hemangiomas [5], presence of ascites [6], presence of coagulopathy [7], use of anticoagulant or antiaggregant drugs [8], type of needle used, and chronic renal failure.

Bleeding can originate from the liver parenchyma, and taking the piece from the mass itself can cause bleeding. Although damage to vascular structures is avoided while performing a biopsy under ultrasound guidance, small-sized vascular structures (especially hepatic artery branches) can be present in the needle route. In addition, biopsies from hypervascular masses can cause more bleeding [9].

The aim of this study was to evaluate the potential new predictors of bleeding based on ultrasonographic features in liver mass biopsies, which are frequently applied in routine clinical practice.

## 2. Materials and methods

This retrospective study was approved by the local ethics committee and conducted in accordance with the Declaration of Helsinki. Informed consent was waived because of the retrospective nature of the study.

The analysis of the images and data of patients who underwent an ultrasonography-guided percutaneous liver mass biopsy was performed at the Interventional Radiology Department of Sakarya University Training and Research Hospital between January 2018 and December 2019; various indications were retrospectively reviewed using the hospital information system. Biopsy patients over the age of 18 years, whose ultrasonography (US) information was available before the procedure and revealed pathological findings, were included in the study. Lesions of vascular origin, such as hemangiomas, were excluded from the study. All patients were evaluated with US by a 7-year experienced radiologist (O.T.) immediately before the procedure. Liver size, liver steatosis status (grade 0–3), parenchyma appearance (homogenous or heterogeneous and coarse granular), vascularity on Doppler US before the procedure (hypo or hypervascular), and hemoglobin (Hb) values before and after the procedure were recorded. Grading due to diffuse hepatic steatosis was performed as follows: If hepatic echogenicity is increased, but periportal and diaphragmatic echogenicity can still be differentiated, it was defined as grade 1; if periportal echogenicity is obscured due to increased hepatic echogenicity, but the diaphragmatic echogenicity can still be differenciated, it was defined as grade 2; if diaphragmatic echogenicity could not be differenciated, it was defined as grade 3. If liver’s craiocaudal diameter is longer than 17 cm in the midclavicular line (MCL), it was considered hepatomegaly. In addition, we divided the patients as hepatocellular carcinoma (HCC) or metastatic disease according to pathological results. In patients whose pathological result showed metastasis, in case the primary metastasis focus could not be detected pathologically, the decision of metastatic disease was made according to the results of radiological, scintigraphy images, and biopsies taken from the primary focus.

Biopsies were performed by an interventional radiologist with 8 years of experience (Ö.F.A.) under US guidance. Before the procedure, routine blood tests and bleeding parameters were requested from the patients. The patients were monitored during the procedure, and 2% prilocaine was used for local anesthesia (Priloc, Vem, Turkey). A US-guided biopsy was performed with a 3.5 MHz convex probe using an Esaote MyLab 50 device (Esaote S.p.A, Italy). Local anesthesia was induced after the site was cleaned. The biopsy was performed with an 18G fully automatic biopsy needle (Geotek, Turkey). The specimens were fixed with formalin.

After the procedure, patients were recommended absolute movement restriction for four hours. After biopsy, Hb was followed up at the third hour and at the sixth hour. Control US was performed for the presence of intraabdominal fluid in patients with Hb decrease. In addition, all patients underwent US for control before discharge.

Statistical analysis

MedCalc (ver. 12, Ostend, Belgium) was used for statistical analysis. Descriptive statistics were given as median (minimum – maximum) and mean ± standard deviation values. Categorical variables were expressed as frequencies and percentages. The independent and paired samples t-test was used for the comparison of continuous variables with normal distribution according to the Kolmogorov–Smirnov and Shapiro–Wilk tests. Nonparametric tests (Mann–Whitney U and Kruskal–Wallis) were used for the comparison of continuous variables due to the lack of normal distribution in the Kolmogorov–Smirnov and Shapiro–Wilk tests. Comparison of parameters before/after biopsy was done with Wilcoxon signed rank test and comparison of parameters to pathological diagnosis that were done with Kruskall–Wallis test according to data distribution. Bonferroni correction was made for comparison number bigger than 3. A value of P < 0.05 was accepted as statistically significant.

## 3. Results

Information on 211 liver mass patients was obtained. Preprocedure US information for 31 of these patients could not be obtained from the hospital’s data system. Four patients were excluded due to the lack of pathological diagnosis in the first biopsy caused by necrosis or insufficient specimen. A total of 176 patients were included in the study. Ninety-six patients were male (54.5%) and 80 were female (45.5%). The mean age of the patients was 64 ± 12.3 years. The pathological diagnosis was HCC in 56 patients (31.8%), colorectal metastasis in 56 (31.8%), lung cancer metastasis in 24 (13.7%), neuroendocrine tumor metastasis in 12 (6.8%), breast cancer metastasis in 12 (6.8%), pancreatic cancer metastasis (6.8%) in 12, and renal cell carcinoma metastasis (2.3%) in four.

**Figure 1:**
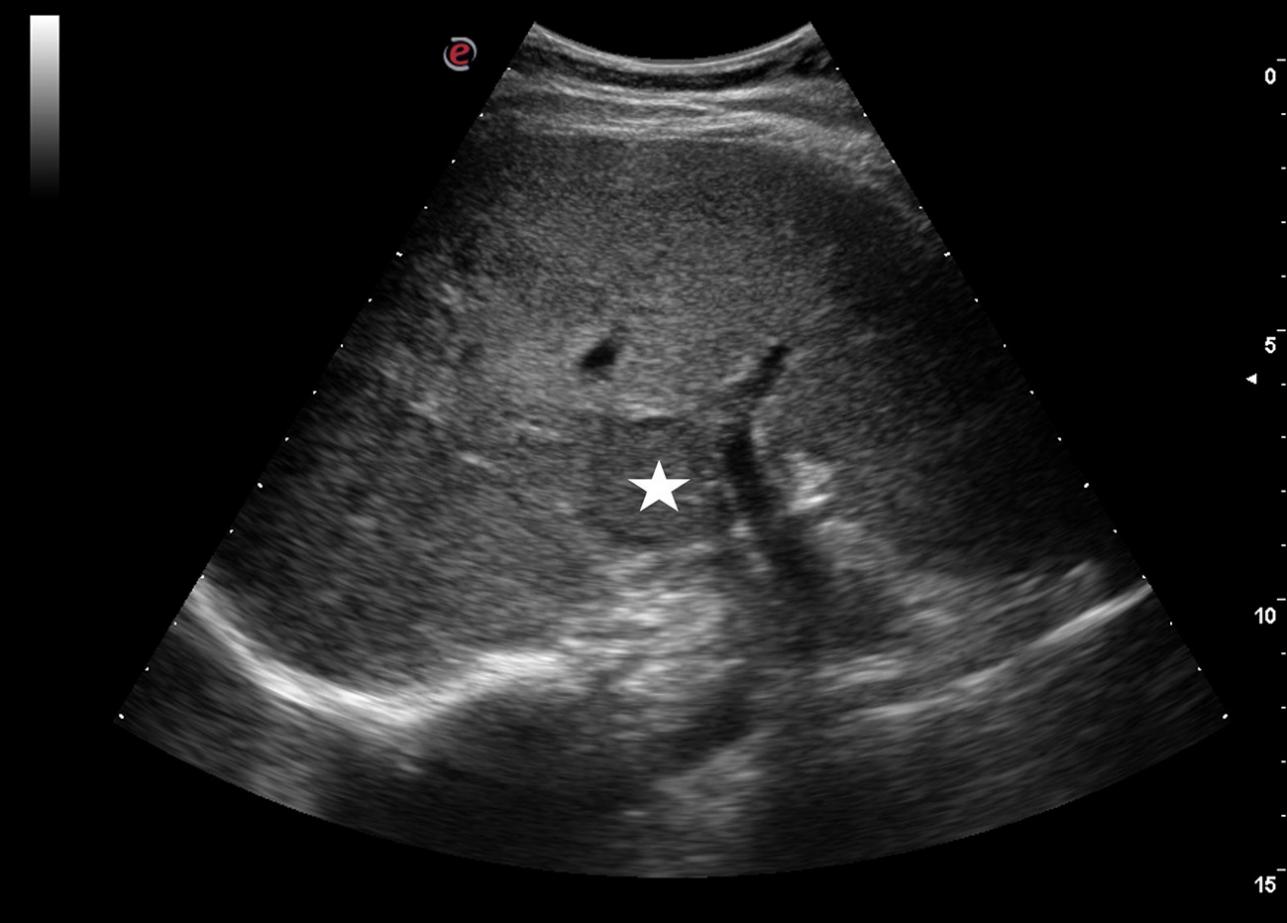
Ultrasound image of a 56-year-old female patient. A focal lesion (asterisk) is observed in the homogeneous liver parenchyma of the patient who is being followed up due to breast cancer.

**Figure 2:**
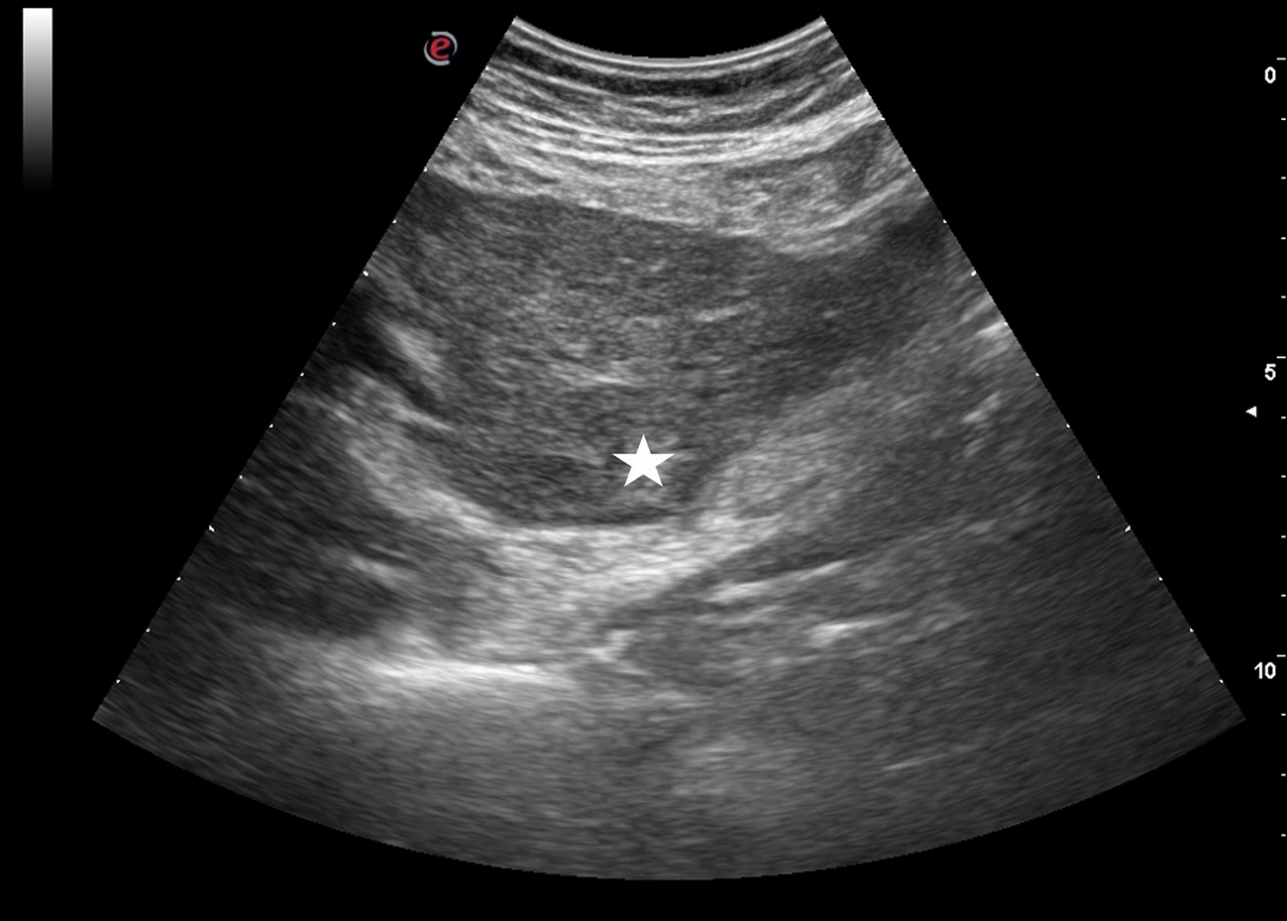
Ultrasound image of a 62-year-old male patient. A focal lesion (asterisk) is observed in the heterogeneous and coarse granular liver parenchyma of the patient who is being followed up for hepatitis B.

Eighty patients (45.5%) had hepatomegaly and 56 had hepatosteatosis (31.8%). In 120 patients (68.8%), the liver parenchyma was observed as homogeneous (Figure 1). In 56 patients, a heterogeneous and coarse granular pattern (Figure 2) was observed in the liver parenchyma (31.8%).

The mean Hb values of the patients were 11.5 ± 1.9 gr/dL before the procedure and 11.4 ± 1.5 gr/dL after the procedure. While 144 of the patients had less than 10% hemoglobin decrease (81.8%), 32 had more than 10% decrease (8.2%). The patients with an Hb decrease of more than 10% did not require treatment during the follow-up, except for one patient. Major bleeding and pseudoaneurysm developed in one patient (0.5%) and was angiographically embolized with particles and coils.

For all patients, no significant difference was found between the Hb values before and after the procedure (P = 0.226). When a subgroup analysis was performed, no significant difference was found in those with a pathological diagnosis of HCC (P = 0.081) and metastases (P = 0.629). In addition, no significant difference was observed in patients with normal liver parenchyma (P = 0.736). However, there was a significant difference between the Hb values measured before and after the procedure in those with a heterogeneous and coarse granular structure of the liver parenchyma (P = 0.012).

The mean Hb decrease in patients with a pathological diagnosis of HCC was 5.4% ± 3.3%, while it was 6.9% ± 4.1% in the group with metastases, with no significant difference between the two groups (P = 0.447) (Table 1). The decrease in the Hb rate (the ratio of change in Hb after the procedure compared to the baseline values) was 6.6 ± 5.3% in patients with normal parenchyma, while it was 8.7% ± 4.9% in those with heterogeneous and coarse granular parenchyma. There was a significant difference between these two groups (P = 0.036). The findings obtained from these patients and related to the appearance of liver parenchyma are summarized in Table 2.

**Table 1:** Hemoglobin values of the study groups before and after procedures by the pathological diagnosis.

	Hepatocellular carcinoma (n = 56)	Metastasis(n = 120)	P*
Hb before biopsy (gr/dL)	11.8 ± 1.4	11.7 ± 1.3	0.542
Hb after biopsy (gr/dL)	11.2 ± 1.6	10.9 ± 1.2	0.318
Hb decrease (%)	5.4 ± 3.3	6.9 ± 4.1	0.447

*Mann–Whitney U test, Hb: hemoglobin

**Table 2:** The comparison of clinical findings according to the parenchyma appearance.

	Normal (n = 120)	Heterogeneous (n = 56)	P*
Gender (M/F)	60/60	36/20	0.445
Age (mean)	59 ± 12.1	63 ± 12.5	0.123
Preprocedural Hb (gr/dL)	11.3 ± 1.6	12 ± 1.4	0.283
Postprocedural Hb (gr/dL)	10.6 ± 1.3	10.9 ± 1.4	0.226
Decrease in Hb (%)	6.6 ± 5.3	8.7 ± 4.9	0.036

*Mann–Whitney U test, Hb: hemoglobin

When the preprocedural Doppler ultrasonography examination of the patients was evaluated, the lesions were hypervascular in 72 patients (40.9%) and hypovascular in 104 patients (59.1%). In terms of the Hb decrease, no significant difference was found between the hypervascular group (6.8%) and the hypovascular group (6.3%) (P = 0.083). Similarly, no significant difference was found in terms of Hb decrease in the patients with and without hepatomegaly (P = 0.075), and those with and without hepatosteatosis (P = 0.954).

## 4. Discussion

In our study, it has been shown that patients with coarse granular pattern in the liver have a higher risk of bleeding due to ultrasound-guided liver mass biopsy compared to patients with homogeneous liver parenchyma. However, in the Doppler ultrasonography evaluation of the target mass which biopsy is performed, it was observed that being hypervascular and hypovascular did not significantly change the risk of bleeding due to the procedure.

A liver mass biopsy is a procedure performed frequently in every interventional radiology clinic due to many indications. Although it is a very reliable method, it is not free of complications, the most common of which is bleeding — a potentially fatal complication.. There are many suggestions and procedures in textbooks and guidelines regarding appropriate patient selection and management to reduce complications [10–12]. The most important of these recommendations include the regulation of anticoagulant and antiaggregant treatments, evaluation of bleeding parameters before the procedure, nonbiopsy from vascular masses unless absolutely necessary, performing fine needle biopsies, and evaluation of patient compatibility. The bleeding complication can be seen in liver mass biopsies despite following all these recommendations and performing appropriate procedures and imaging. For this reason, it is very important to know in advance any situation that may increase the risk of bleeding, to prevent complications if possible, and to be prepared for an intervention when a complication occurs [13]. In our study, we compared the patients with homogeneous liver parenchyma or a coarse granular pattern and heterogeneous liver parenchyma in patients who were evaluated by US before biopsy, and the Hb decrease in the coarse granular pattern was found to be significantly higher (P = 0.012). As the needle reaches the target lesion, it may pass through the diseased liver parenchyma or nontarget mass along its route. In the route, where the needle passes, bleeding may originate from any location. Another possibility is that the tamponade effect may not be sufficient in a diseased liver. Although Solbiati et al. suggest passing 1 to 2 cm normal hepatic parenchyma before entering the target lesion while performing percutaneous liver biopsies [14], no significant relationship was found between the depth of the lesion and bleeding in a recent study by Potretzke et al [9]. In a prospective study conducted by Prud’homme et al., the most important risk factors for complications after biopsy were found as biopsy location, higher number of collected samples, and lesion size [15]. Although the study does not contain any information about liver parenchyma, especially higher number of collected samples and lesion size parameters show the importance of buffering effect.

Due to the tamponade effect of the liver parenchyma, it is recommended to perform the biopsy from the deeply located lesion or passing through the liver parenchyma as much as possible in subcapsular lesions. Although the structure of a normal liver is suitable for the tamponade effect, pathological liver parenchyma is more fragile; thus, it may not have enough tamponade effect, or the fragile structure may predispose the liver parenchyma to bleeding [13,14]. In addition, the fragility of the mass may be higher than other masses due to mass-induced hypervascularity, especially in lesions that are enhanced in the arterial phase (with high arterial supply) due to cutting during biopsy.

In this study, hypervascular masses were also compared. Although the Hb decrease was slightly higher in the biopsies taken from the hypervascular lesions compared to the hypovascular lesions (6.8% and 6.3%, respectively), the difference between the two groups was not significant (P = 0.083). This result shows that unless the lesion has a vascular origin (such as hemangiomas), the nature of the mass examined before the procedure is not decisive in terms of Hb decrease.

In the comparison of patients with and without hepatomegaly and those with and without hepatosteatosis, there was no significant difference in bleeding. Therefore, the presence of these findings before the biopsy does not pose a risk for bleeding. In our study, the major bleeding rate was 0.5% (1/176), which is consistent with previous studies [16–19]. There was no death due to biopsy.

Besides being a very important diagnostic tool in the diagnosis of liver masses, ultrasonography is also widely used in terms of biopsy guidance. In our study, it was shown that besides this importance of ultrasound, it can predict the risk of bleeding in percutaneous liver mass biopsies. Being able to make an ultrasonographic risk estimation for bleeding before the biopsy procedure will lead the practitioner to be more careful and take precautions during the patient preparation, during the application and after the procedure.

Our study has some important limitations. First, it has a retrospective and single-center design. Second, the study group was heterogeneous. In addition, the Hb values ​​of the patients were measured before biopsy, three and six hours after the procedure, and the data on late bleeding were not reported. However, studies have shown that bleeding after biopsy mostly occurs in the first six hours, and almost all bleeding cases present within the first 24 hours with late bleeding being rarely been reported [16,19]. Another limitation of the study concerns the low number of patients.

In conclusion, in our study, it was shown for the first time in the literature that, in addition to other causes that increase the risk of bleeding, the evaluation of the liver parenchyma by US before the biopsy procedure is important in evaluation of the risk of biopsy related bleeding. It will be beneficial for patient management to know that the risk of bleeding is higher in patients whose liver parenchyma is observed in heterogeneous and coarse granular pattern on the US before the procedure.

## Informed Consent

This retrospective study has been approved by the local ethics committee (Sakarya University, 71522473/050.01.04/40) and conducted in accordance with the Declaration of Helsinki. Informed consent was waived because of the retrospective nature of the study.
